# SAGER: a database of Symbiodiniaceae and Algal Genomic Resource

**DOI:** 10.1093/database/baaa051

**Published:** 2020-07-04

**Authors:** Liying Yu, Tangcheng Li, Ling Li, Xin Lin, Hongfei Li, Chichi Liu, Chentao Guo, Senjie Lin

**Affiliations:** 1 State Key Laboratory of Marine Environmental Science and College of Ocean and Earth Sciences, Xiamen University, Xiamen 361102, China; 2 Department of Marine Sciences, University of Connecticut, Groton, CT 06340, USA

## Abstract

Symbiodiniaceae dinoflagellates are essential endosymbionts of reef building corals and some other invertebrates. Information of their genome structure and function is critical for understanding coral symbiosis and bleaching. With the rapid development of sequencing technology, genome draft assemblies of several Symbiodiniaceae species and diverse marine algal genomes have become publicly available but spread in multiple separate locations. Here, we present a Symbiodiniaceae and Algal Genomic Resource Database (SAGER), a user-friendly online repository for integrating existing genomic data of Symbiodiniaceae species and diverse marine algal gene sets from MMETSP and PhyloDB databases. Relevant algal data are included to facilitate comparative analyses. The database is freely accessible at http://sampgr.org.cn. It provides comprehensive tools for studying gene function, expression and comparative genomics, including search tools to identify gene information from Symbiodiniaceae species, and BLAST tool to find orthologs from marine algae and protists. Moreover, SAGER integrates transcriptome datasets derived from diverse culture conditions of corresponding Symbiodiniaceae species. SAGER was developed with the capacity to incorporate future Symbiodiniaceae and algal genome and transcriptome data, and will serve as an open-access and sustained platform providing genomic and molecular tools that can be conveniently used to study Symbiodiniaceae and other marine algae.

Database URL: http://sampgr.org.cn

## Introduction

Symbiodiniaceae, symbiotic dinoflagellates, are well known as essential endosymbionts of reef building corals and some other invertebrates (1). They vary in the diversity and abundance with different hosts and environments (1–3). For example, the dominant Symbiodiniaceae species can shuffle during the process of coral bleaching (4, 5). Under environmental stress conditions, Symbiodiniaceae are expelled from their hosts resulting in coral bleaching (6). In face of stress resulting from climate change and anthropogenic disturbance, there has been increasingly widespread and severe coral degradation in recent decades, largely due to the disruption of the coral-dinoflagellate symbiosis (i.e. coral bleaching) (7, 8). To understand molecular mechanisms underpinning symbiosis and its disruption and develop strategies to conserve coral reefs, there have been dedicated efforts employing the high-throughput “omics” technologies to identify genomic and genetic elements associated with these processes (9–13). For example, studies have shown that Symbiodiniaceae evolutionarily expanded genes functioning in nutrient uptake (14), transmembrane transport, and combat of reactive oxygen species and UV radiations (9).

Next generation sequencing has benefited Symbiodiniaceae genomic and transcriptomic studies. So far, eight assemblies of six Symbiodiniaceae species have been reported, including the genome of *Breviolum minutum* (LaJeunesse, J.E.Parkinson & J.D.Reimer) J.E.Parkinson & LaJeunesse (formerly Clade B, *Symbiodinium minutum*) (15), *Fugacium kawagutii* (Trench & R.J.Blank ex LaJeunesse) LaJeunesse (formerly Clade F, *S. kawagutii*) (14, 16, 17), *S. microadriaticum* LaJeunesse (formerly Clade A) (18)*, Cladocopium goreaui* (Trench & R.J.Blank ex LaJeunesse) LaJeunesse & H.J.Jeong (formerly Clade C, *S. goreaui*) (16), *Symbiodinium* sp. Trench & R.J.Blank ex LaJeunesse (formerly Clade A3) and *Cladocopium* sp. (Trench & R.J.Blank ex LaJeunesse) LaJeunesse & H.J.Jeong (formerly Clade C92) (19)*.* However, these resources have not been integrated into a centralized database furnished with analysis tools that is publicly available for comparative genomics and other symbiosis-related studies.

In this paper, we present the Symbiodiniaceae and Algal Genomic Resource Database (SAGER), which integrates Symbiodiniaceae genome resources and provides homology search tools for marine algae and protists. SAGER is aimed to serve as a data resource and user-friendly platform for coral and algal research, and to facilitate comparative genomic analyses. Along with the database, tools such as keyword search, BLAST (20, 21), JBrowse (22) and download will allow Symbiodiniaceae and marine alga researchers to perform various tasks including manual check of gene model annotation and obtaining updates of marine algal genomic resources.

## Database overview

The SAGER integrates Symbiodiniaceae genomic and transcriptomic datasets. This includes assembled genomes, CDS and amino acid sequences, re-annotated gene annotation and gene expression from different culture conditions of six Symbiodiaceae species (*B. minutum*, *F. kawagutii*, *S. microadriaticum*, *C. goreaui*, *Symbiodinium* sp. and *Cladocopium* sp). Keyword search using gene ID, located scaffold, and ID number or any keywords from functional annotation can facilitate users finding target genes in batch. These data can also be downloaded easily and explored online using JBrowse and BLAST tools. The interaction of the various tool pages facilitates researchers to study gene function, expression and comparative genomics. In addition, to meet the needs for comparative studies, the database also integrates two other existing marine plankton genomic resource database – Marine Microbial Eukaryotic Transcriptome Sequencing Project (MMETSP) database (23, 24) & PhyloDB (version 1.076), which can also be downloaded or explored via homology search with BLAST tool.

## Database organization

The SAGER is an extensive open-access database that houses Symbiodiniaceae and marine algal genomic data. It was developed in a user-friendly and online mode, consisting of Search, BLAST, JBrowse and interlinked Gene Information components.

## Gene information

**Figure 1:**
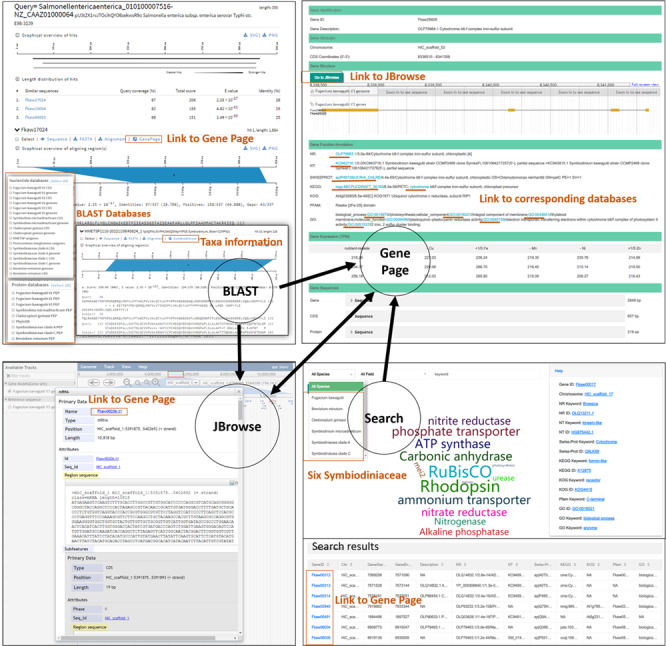
Database organization.

The Gene Information section acts as a core linking component within SAGER, which can be accessed through Search, BLAST and JBrowse (Figure 1). This component displays information for each of the genes from all six Symbiodiniaceae species included in this work, including gene location, gene structure, gene description based on NR annotation, GO and Swiss-Prot annotation, gene expression under different culture conditions, sequences of gene, CDS and amino acid in FASTA format. The most recently available genome version 3 of *F. kawagutii* also includes annotations from databases of NT, KEGG pathway, eggnog (KOG) and PFAM. To better visualize gene structure, full-screen view of gene structure in Gene Information component is linked to JBrowse. Additionally, accession numbers included in the annotation information are linked to their corresponding source databases: NR, NT, Swiss-Prot, GO and KEGG.

## Search

The Search component allows users to retrieve gene information with keywords. The history of searched words is recorded, updated real time, and displayed in word cloud image in the middle of search page. The font size of the displayed word is proportional to the number of times the word has been searched for. Gene ID, scaffold ID, the functional description and accession number from NR, GO and Swiss-Psrot databases of genes from all Symbiodiniaceae species are provided in the Search component. For *F. kawagutii* genome information, users can search for genes using any keywords including accession number and functional annotations from other resources such as KEGG, NT, KOG and PFAM. The search results displayed include all returned genes with their information in list format which can be alternatively selected or filtered for download. The Detail in the column of the search results is linked to Gene Information.

## BLAST server

To facilitate sequence similarity search, we integrated the SequenceServer (http://sequenceserver.com/) (25) into SAGER to enable BLAST analysis (20, 21) and visually display BLAST results. This tool displays alignment results in visual graphics which can be downloaded in SVG and PNG formats. It also provides direct download of sequence and alignment from the result page. Diverse datasets are provided in the BLAST server, including all Symbiodiniaceae genome assembly sequences and the two marine plankton genomic resources – augmented MMETSP (23, 24) and PhyloDB databases. To determine organism source of matched subject gene from MMETSP and PhyloDB, we developed a set of scripts using Ruby and Linux shell programming to list taxon information under the line of subject gene ID at the BLAST output page. This allows users to easily track the taxonomic affiliation of the source organism of the target gene. In addition, the subject ID from Symbiodiniaceae species on the result page can be linked to the corresponding Gene Information page.

**Table 1:** Publicly available genomes of Symbiodiniaceae

**Species**	**Strain**	**Isolation source**	**Clade**	**Assembly size (Mbp)**	**Gene No.**	**Gene symbol prefix**	**Reference**
*B. minutum*	Mf1.05b	Florida Keys	B	616	41 925	symbB	(15)
*F. kawagutii*	CCMP2468, CS-156	Hawaiian	F	935	36 850	Skaw	(14)
				1050	26 609	SymbF	(16)
				937	45 192	Fkaw	(17)
*S. microadriaticum*	CCMP2467	Gulf of Aqaba	A	808	49 109	Smic	(18)
*C. goreaui*	CCMP2466, SCF055	Magnetic Island	C1	1030	35 913	SymbC1	(16)
*Symbiodinium* sp.	Y106	Okinawa	A3	767	71 632	SymA3	(19)
*Cladocopium* sp.	Y103	Okinawa	C92	705	68 732	SymC	(19)

## Genome visualization

Symbiodiniaceae genomes are displayed using JBrowse, a tool designed for genome information visualization (22). We also integrated gene expression evidence of *F. kawagutii* version 3 from a transcriptome of mixed samples from different trace metal conditions. Users can conveniently view genomic scaffolds at any sequence location. This enables users to simultaneously view gene sets and gene expression evidence for manually checking gene structure.

## Download page

In the Download page, Symbiodiniaceae genome datasets and metadata of the augmented MMETSP and PhyloDB are available there for users to download. The downloadable genome resources include genome assembly, GFF3 file, protein and CDS sequences, gene annotation files and gene expression from transcriptomes. The word ‘reference’ is linked to their corresponding publications.

## Database sources

The SAGER database incorporates data of Symbiodiniaceae species and marine algae including: ① genomes (Table 1) and transcriptomes (Table S1) of six Symbiodiniaceae species including *B. minutum* (strain Mf1.05b, isolated from Florida Keys) (15, 26), *F. kawagutii* (strain CCMP2468, isolated from Hawaiian) (14, 16, 17, 27, 28), *S. microadriaticum* (strain CCMP2467, isolated from Gulf of Aqaba) (18, 29), *C. goreaui* (strain CCMP2466, isolated from Magnetic Island) (11, 16), *Symbiodinium* sp. (strain Y106, isolated from Okinawa) and *Cladocopium* sp. (strain Y103, isolated from Okinawa) (19); ② *de novo* transcriptome assembly of 302 marine microeukaryotic species from the MMETSP database (23, 24) and four dinoflagellates from our laboratory involving *Prorocentrum shikokuense* Hada (*P. donghaiense* Lu)*, Karenia mikimotoi* (Miyake & Kominami ex Oda) Gert Hansen & Moestrup (30)*, Effrenium* sp. (Trench & R.J.Blank ex LaJeunesse) LaJeunesse & H.J.Jeong (formerly Clade E) and *Karlodinium veneficum* (D.Ballantine) J.Larsen; and ③ Protein sequences of marine plankton from PhyloDB (version 1.076), which includes Eukaryota, Bacteria, Archaea and Viruses, and above-mentioned four dinoflagellates.

## Symbiodiniaceae genome assemblies and transcriptomes

The first draft assembly of Symbiodiniaceae species was the genome of *B. minutum* (15), which was followed by that of *F. kawagutii* (14, 16, 17), *S. microadriaticum* (18)*, C. goreaui* (16), *Symbiodinium* sp. and *Cladocopium* sp. (19) (Table 1)*.* Among them, *F. kawagutii* has been updated after the initial release in 2015. Following its first revision (14), the second revision has just been published (16). In this version 3 (17), the genome assembly showed a N50 > 13 Mbp and the longest scaffold of 121 Mb, likely complete or nearly complete chromosome, and the number of predicted genes increased from 36 850 to 45 192. This is so far the best assembled genome of Symbiodiniaceae. Except *F. kawagutii* genes that already had the most recent annotation information, reannotation was carried out for all Symbiodiniaceae genes using Diamond blastx (E value =1E-5) to search against NCBI NR (updated on June 30, 2019) and Swiss-Prot databases (updated on March 20, 2020). And Blast2GO (31) was used to obtain the Gene Ontology (GO) annotation based on NR annotation. For those genes which have multiple transcript sequences, we selected the longest one to represent the gene in CDS and amino acid sequence files.

We also integrated published transcriptomic data of six Symbiodiniaceae species into SAGER (Table S1). Gene expression of each transcriptome was calculated based on raw sequencing data using same methods. Firstly, raw next-generation sequencing data were downloaded from the NCBI SRA database. Secondly, quality trimming was conducted on raw reads to remove poor quality data using Trimmomatic (32) with parameters setting as: LEADING:5 TRAILING:5 SLIDINGWINDOW:4:15 MINLEN:50. Finally, the trimmed clean reads were mapped to corresponding genome reference using Bowtie2 (33) and counted using RSEM software (34). Gene expression was normalized as Transcripts Per Million (TPM), and averaged across biological replicates. Totally, there are 66 transcriptomes of six Symbiodiaceae from different culture conditions (Table S1). *B. minutum* transcriptome was from cultures grown under normal conditions (L1 medium, 26°C) (26). Transcriptomes of *F. kawagutii* were from cultures grown under normal conditions (L1 medium, 25°C), heat stress, phosphate deprivation, organic phosphorus (OP) as P-source (Gro3P replacement) (27), and different trace metal conditions, including normal (L1 medium, 26°C) and reduced concentrations of Cu, Fe, Mn, Ni and Zn (28). *S. microadriaticum* transcriptomes represent samples grown under cold shock, cold stress, heat stress, heat shock, hyposalinity, hypersalinity, dark stress, dark cycle and control (12 h/12 h day/night cycle, 23°C) conditions (29). *C. goreaui* transcriptomes were from samples collected under control culture condition (27°C) on day 1, 32°C on day 9, 27°C on day 9, 32°C on day 13 and 27°C on day 13 (11). *Symbiodinium* sp. and *Cladocopium* sp. each has five transcriptomes under control culture condition (0 h and 48 h, 25°C), dark (48 h), heat stress (48 h) and heat stress in the dark (48 h) (19).

**Figure 2:**
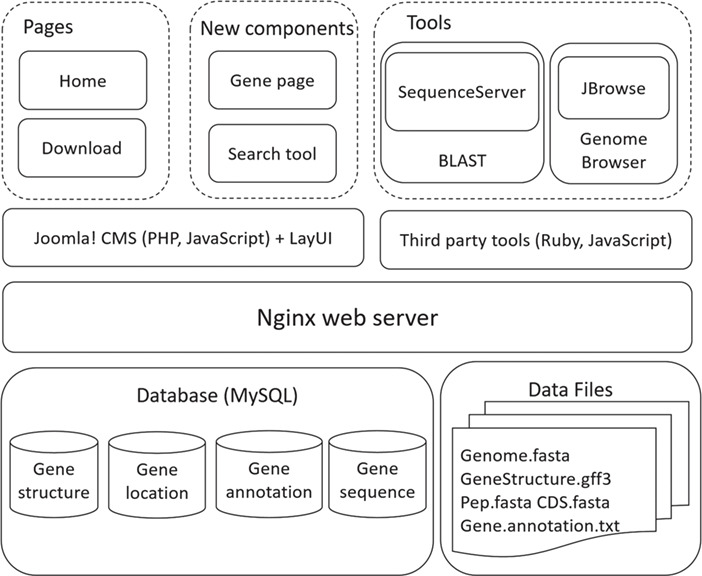
Architecture of SAGER.

## Marine phytoplankton transcriptome *de novo* assemblies

The MMETSP dataset contains transcriptomes from samples of marine eukaryotes representing more than 40 phyla (24). In SAGER, we integrated 678 transcriptomic *de-novo* re-assemblies from MMESTP representing 302 marine phytoplankton species (23). In addition, four dinoflagellate transcriptomes not covered in MMETSP that were sequenced in our laboratory were also included to expand species diversity, involving *P. shikokuense* (unpublished)*, K. mikimotoi* (30)*, Effrenium* sp. (unpublished) and *K. veneficum* (unpublished). *P. shikokuense* transcriptomes include non-redundant unigenes that merged with transcriptomic assemblies of culture samples collected at cell cycle phases S, M, G1 and G2, and grown under conditions of nitrogen (N)-replete, N-depleted, phosphorus (P)-depleted and P- replete using CD-HIT (35). *K. mikimotoi* unigenes were differentially expressed genes profiled from cultures grown in L1 medium (+P) and ATP-replacing-DIP medium (ATP) compared with DIP-depleted L1 medium (–P) using suppression subtractive hybridisation (SSH) followed by 454 pyrosequencing (30). *Effrenium* sp. transcriptomes combined unigenes data incorporating cultures grown at 20°C, 26°C and 33°C. Unigenes of *K. veneficum* were combination of splice leader-based transcriptomes of cultures grown in L1 medium and under mixotrophic condition, sampled during day (illuminated) and night (dark) time points. These resulted in 682 transcriptomic assembly of 306 phytoplankton species, containing 32 677 544 unigenes. These unigenes were annotated using Diamond blastx (E value =1E-5) against NCBI NR database (updated on June 30, 2019). Among them, 11 947 623 (36.56%) had functional annotation.

## Marine plankton protein dataset

PhyloDB is a database suitable for comprehensive annotation of metagenomics and metatranscriptomics analyses (36), which is comprised of protein sequences from KEGG, GenBank, JGI, ENSEMBL, and initial assembled MMETSP databases (24). This dataset (version 1.076) was downloaded from https://scripps.ucsd.edu/labs/aallen/data/ (see “Databases and Collections”). Although the MMETSP assemblies in PhyloDB were older than the above re-assembled version, we still keep the old version in PhyloDB to maintain the species diversity in this dataset. However, we augmented the dataset by incorporating the protein sequences predicted from the above-mentioned four additional datasets of dinoflagellate unigenes using TransDecoder v5.5.0 (https://github.com/TransDecoder/TransDecoder/releases). As a result, the PhyloDB in SAGER consists of 29 690 621 protein sequences of 25 996 species, including 894 of Eukaryota, 4910 of Bacteria, 230 of Archaea and of 19 962 Viruses.

## System implementation

SAGER was developed with combination of several tools and scripts (Figure 2). Genomic data were imported and managed with MySQL. The Nginx web server was used to construct the underlying web server. Tools of Joolma! Content Management System (CMS) and LayUI were used to build home and download pages, and new components of gene page and search tool. Third party tools of SequenceServer (25) and JBrowse (22) were used to construct BLAST and Genome Browser tools. PHP and JavaScript were applied to make the pages flexible and interactive.

## Future work

SAGER is aimed to be a user-friendly database and tool resource, which integrates currently available Symbiodiniaceae genome data, marine phytoplankton genome resources, and analysis as well as visualization tools. It is worth noting that we used Symbiodiniaceae genomic resources that were generated by different research groups using slightly different approaches. Therefore, users of our integrated data should keep in mind that variations are likely to occur between datasets, which will affect comparative analyses between Symbiodiniaceae genomes, as recently demonstrated (37). Furthermore, the database was designed with room to accommodate and house newly generated data. We will continue to update and upgrade the data resources. Future updates will cover transcriptomes of Symbiodiniaceae and relevant cultured marine phytoplankton and field-collected samples such as coral holobiont (38–40) or harmful algal bloom metatranscriptomes (41, 42). To better support the capability of SAGER to serve the research community, new web tools will be developed to allow more efficient and effective use of this database.

## Supplementary Material

Table_S1
