# Supplementary material for: SAGER: a database of Symbiodiniaceae and Algal Genomic Resource
Source: Database (Oxford). 2020 Jul 4;2020:baaa051. doi: 10.1093/database/baaa051 (PMC7334889; doi:10.1093/database/baaa051)
Supplement: Table_S1 [file table_s1.docx]

**Table S1. Publicly available transcriptomes of Symbiodiniaceae.**

| Symbiodiniaceae (ref) | **Sample name (condition)** | **Data size (MB)** | **Read length (bp)** | **Read bases (Gbp)** | **Accession number** | **Raw data download site** |
| --- | --- | --- | --- | --- | --- | --- |
| *B. minutum* (26) | Normal (L1 medium, 26℃) | 1683 | 2 x 101 | 3.89 | SRR1793320 | https://sra-downloadb.be-md.ncbi.nlm.nih.gov/sos1/sra-pub-run-2/SRR1793320/SRR1793320.1 |
| *F. kawagutii* (27, 28) | Heat stress (30℃) | 960 | 2 x 50 | 1.51 | SRR1300302 | https://sra-downloadb.be-md.ncbi.nlm.nih.gov/sos1/sra-pub-run-5/SRR1300302/SRR1300302.2 |
|  | Normal (L1 medium, 25℃) | 701 | 2 x 50 | 1.11 | SRR1300303 | https://sra-downloadb.be-md.ncbi.nlm.nih.gov/sos1/sra-pub-run-5/SRR1300303/SRR1300303.2 |
|  | P deprived | 994 | 2 x 50 | 1.58 | SRR1300304 | https://sra-downloadb.be-md.ncbi.nlm.nih.gov/sos1/sra-pub-run-5/SRR1300304/SRR1300304.2 |
|  | Gro3P replacing DIP | 1571 | 2 x 50 | 2.47 | SRR1300305 | https://sra-downloadb.be-md.ncbi.nlm.nih.gov/sos1/sra-pub-run-5/SRR1300305/SRR1300305.2 |
|  | Control (L1 medium, 26℃) | 712 | 1 x 50 | 1.20 | SRR9417744 | https://sra-download.ncbi.nlm.nih.gov/traces/sra61/SRR/009197/SRR9417755 |
|  | Control (L1 medium, 26℃) | 702 | 1 x 50 | 1.21 | SRR9417745 | https://sra-download.ncbi.nlm.nih.gov/traces/sra73/SRR/009197/SRR9417756 |
|  | Control (L1 medium, 26℃) | 701 | 1 x 50 | 1.21 | SRR9417746 | https://sra-download.ncbi.nlm.nih.gov/traces/sra5/SRR/009197/SRR9417753 |
|  | -Cu | 702 | 1 x 50 | 1.21 | SRR9417747 | https://sra-download.ncbi.nlm.nih.gov/traces/sra0/SRR/009197/SRR9417754 |
|  | -Cu | 702 | 1 x 50 | 1.21 | SRR9417748 | https://sra-download.ncbi.nlm.nih.gov/traces/sra2/SRR/009197/SRR9417751 |
|  | -Cu | 697 | 1 x 50 | 1.21 | SRR9417749 | https://sra-download.ncbi.nlm.nih.gov/traces/sra61/SRR/009197/SRR9417752 |
|  | +1/5 Fe | 699 | 1 x 50 | 1.21 | SRR9417750 | https://sra-download.ncbi.nlm.nih.gov/traces/sra76/SRR/009197/SRR9417749 |
|  | +1/5 Fe | 697 | 1 x 50 | 1.21 | SRR9417751 | https://sra-download.ncbi.nlm.nih.gov/traces/sra41/SRR/009197/SRR9417750 |
|  | +1/5 Fe | 715 | 1 x 50 | 1.21 | SRR9417752 | https://sra-download.ncbi.nlm.nih.gov/traces/sra44/SRR/009197/SRR9417757 |
|  | -Mn | 703 | 1 x 50 | 1.20 | SRR9417753 | https://sra-download.ncbi.nlm.nih.gov/traces/sra16/SRR/009197/SRR9417758 |
|  | -Mn | 714 | 1 x 50 | 1.21 | SRR9417754 | https://sra-download.ncbi.nlm.nih.gov/traces/sra42/SRR/009197/SRR9417747 |
|  | -Mn | 700 | 1 x 50 | 1.21 | SRR9417755 | https://sra-download.ncbi.nlm.nih.gov/traces/sra16/SRR/009197/SRR9417748 |
|  | -Ni | 702 | 1 x 50 | 1.21 | SRR9417756 | https://sra-download.ncbi.nlm.nih.gov/traces/sra2/SRR/009197/SRR9417744 |
|  | -Ni | 699 | 1 x 50 | 1.21 | SRR9417757 | https://sra-download.ncbi.nlm.nih.gov/traces/sra61/SRR/009197/SRR9417759 |
|  | -Ni | 696 | 1 x 50 | 1.21 | SRR9417758 | https://sra-download.ncbi.nlm.nih.gov/traces/sra44/SRR/009197/SRR9417760 |
|  | +1/5 Zn | 695 | 1 x 50 | 1.21 | SRR9417759 | https://sra-download.ncbi.nlm.nih.gov/traces/sra5/SRR/009197/SRR9417745 |
|  | +1/5 Zn | 699 | 1 x 50 | 1.21 | SRR9417760 | https://sra-download.ncbi.nlm.nih.gov/traces/sra77/SRR/009197/SRR9417746 |
|  | +1/5 Zn | 721 | 1 x 50 | 1.21 | SRR9417761 | https://sra-download.ncbi.nlm.nih.gov/traces/sra47/SRR/009197/SRR9417761 |
| *S. microadriaticum* (29) | Cold shock (4℃ for 4 h) | 3229 | 2 x 101 | 5.28 | [SRR867593](https://trace.ncbi.nlm.nih.gov/Traces/sra/?run=SRR867593) | https://sra-downloadb.be-md.ncbi.nlm.nih.gov/sos1/sra-pub-run-2/SRR867593/SRR867593.2 |
|  | Cold stress (16℃ for 4 h) | 4263 | 2 x 101 | 6.97 | [SRR867594](https://trace.ncbi.nlm.nih.gov/Traces/sra/?run=SRR867593) | https://sra-downloadb.be-md.ncbi.nlm.nih.gov/sos1/sra-pub-run-2/SRR867594/SRR867594.2 |
|  | Heat stress (34℃ for 12 h) | 5177 | 2 x 101 | 8.47 | [SRR867595](https://trace.ncbi.nlm.nih.gov/Traces/sra/?run=SRR867593) | https://sra-downloadb.be-md.ncbi.nlm.nih.gov/sos1/sra-pub-run-2/SRR867595/SRR867595.2 |
|  | Heat shock (36℃ for 4 h) | 3805 | 2 x 101 | 6.20 | [SRR867596](https://trace.ncbi.nlm.nih.gov/Traces/sra/?run=SRR867593) | https://sra-downloadb.be-md.ncbi.nlm.nih.gov/sos1/sra-pub-run-2/SRR867596/SRR867596.2 |
|  | Hyposalinity (20 g/L NaCl salt content for 4 h) | 4841 | 2 x 101 | 7.92 | [SRR867597](https://trace.ncbi.nlm.nih.gov/Traces/sra/?run=SRR867593) | https://sra-downloadb.be-md.ncbi.nlm.nih.gov/sos1/sra-pub-run-2/SRR867597/SRR867597.2 |
|  | Hypersalinity (60 g/L NaCl salt content for 4 h) | 4319 | 2 x 101 | 7.05 | [SRR867598](https://trace.ncbi.nlm.nih.gov/Traces/sra/?run=SRR867593) | https://sra-downloadb.be-md.ncbi.nlm.nih.gov/sos1/sra-pub-run-2/SRR867598/SRR867598.2 |
|  | Dark stress (18 h dark period) | 4575 | 2 x 101 | 7.43 | [SRR867599](https://trace.ncbi.nlm.nih.gov/Traces/sra/?run=SRR867593) | https://sra-downloadb.be-md.ncbi.nlm.nih.gov/sos1/sra-pub-run-2/SRR867599/SRR867599.2 |
|  | Dark cycle (12 h/12 h day/night cycle, sampled at midnight) | 3559 | 2 x 101 | 5.82 | [SRR867600](https://trace.ncbi.nlm.nih.gov/Traces/sra/?run=SRR867593) | https://sra-downloadb.be-md.ncbi.nlm.nih.gov/sos1/sra-pub-run-2/SRR867600/SRR867600.2 |
|  | Control (12 h/12 h day/night cycle, 23℃, sampled at noon). | 3702 | 2 x 101 | 6.06 | [SRR867601](https://trace.ncbi.nlm.nih.gov/Traces/sra/?run=SRR867593) | https://sra-downloadb.be-md.ncbi.nlm.nih.gov/sos1/sra-pub-run-2/SRR867601/SRR867601.2 |
| *C. goreaui* (11) | Control (27℃ on day 1) | 331 | 1 x 100 | 1.02 | SRR2298870 | https://sra-downloadb.be-md.ncbi.nlm.nih.gov/sos1/sra-pub-run-1/SRR2298870/SRR2298870.1 |
|  | Control (27℃ on day 1) | 302 | 1 x 100 | 0.93 | SRR2298871 | https://sra-downloadb.be-md.ncbi.nlm.nih.gov/sos1/sra-pub-run-1/SRR2298871/SRR2298871.1 |
|  | Control (27℃ on day 1) | 315 | 1 x 100 | 0.97 | SRR2298872 | https://sra-downloadb.be-md.ncbi.nlm.nih.gov/sos1/sra-pub-run-1/SRR2298872/SRR2298872.1 |
|  | Control (27℃ on day 1) | 312 | 1 x 100 | 0.96 | SRR2298873 | https://sra-downloadb.be-md.ncbi.nlm.nih.gov/sos1/sra-pub-run-1/SRR2298873/SRR2298873.1 |
|  | Control (27℃ on day 1) | 327 | 1 x 100 | 1.01 | SRR2298874 | https://sra-downloadb.be-md.ncbi.nlm.nih.gov/sos1/sra-pub-run-1/SRR2298874/SRR2298874.1 |
|  | Control (27℃ on day 1) | 324 | 1 x 100 | 1.00 | SRR2298875 | https://sra-downloadb.be-md.ncbi.nlm.nih.gov/sos1/sra-pub-run-1/SRR2298875/SRR2298875.1 |
|  | Control (27℃ on day 1) | 324 | 1 x 100 | 1.00 | SRR2298876 | https://sra-downloadb.be-md.ncbi.nlm.nih.gov/sos1/sra-pub-run-1/SRR2298876/SRR2298876.1 |
|  | Control (27℃ on day 1) | 316 | 1 x 100 | 0.97 | SRR2298877 | https://sra-downloadb.be-md.ncbi.nlm.nih.gov/sos1/sra-pub-run-1/SRR2298877/SRR2298877.1 |
|  | 32℃ on day 9 | 315 | 1 x 100 | 0.98 | SRR2298878 | https://sra-downloadb.be-md.ncbi.nlm.nih.gov/sos1/sra-pub-run-1/SRR2298878/SRR2298878.1 |
|  | 32℃ on day 9 | 344 | 1 x 100 | 1.06 | SRR2298879 | https://sra-downloadb.be-md.ncbi.nlm.nih.gov/sos1/sra-pub-run-1/SRR2298879/SRR2298879.1 |
|  | 32℃ on day 9 | 323 | 1 x 100 | 1.00 | SRR2298882 | https://sra-downloadb.be-md.ncbi.nlm.nih.gov/sos1/sra-pub-run-1/SRR2298882/SRR2298882.1 |
|  | 32℃ on day 9 | 322 | 1 x 100 | 0.99 | SRR2298883 | https://sra-downloadb.be-md.ncbi.nlm.nih.gov/sos1/sra-pub-run-1/SRR2298883/SRR2298883.1 |
|  | 27℃ on day 9 | 323 | 1 x 100 | 0.99 | SRR2298880 | https://sra-downloadb.be-md.ncbi.nlm.nih.gov/sos1/sra-pub-run-1/SRR2298880/SRR2298880.1 |
|  | 27℃on day 9 | 320 | 1 x 100 | 0.99 | SRR2298881 | https://sra-downloadb.be-md.ncbi.nlm.nih.gov/sos1/sra-pub-run-1/SRR2298881/SRR2298881.1 |
|  | 27℃ on day 9 | 322 | 1 x 100 | 0.99 | SRR2298884 | https://sra-downloadb.be-md.ncbi.nlm.nih.gov/sos1/sra-pub-run-1/SRR2298884/SRR2298884.1 |
|  | 27℃ on day 9 | 304 | 1 x 100 | 0.94 | SRR2298885 | https://sra-downloadb.be-md.ncbi.nlm.nih.gov/sos1/sra-pub-run-1/SRR2298885/SRR2298885.1 |
|  | 32℃ on day 13 | 312 | 1 x 100 | 0.94 | SRR2298886 | https://sra-downloadb.be-md.ncbi.nlm.nih.gov/sos1/sra-pub-run-1/SRR2298886/SRR2298886.1 |
|  | 32℃ on day 13 | 315 | 1 x 100 | 0.98 | SRR2298887 | https://sra-downloadb.be-md.ncbi.nlm.nih.gov/sos1/sra-pub-run-1/SRR2298887/SRR2298887.1 |
|  | 32℃ on day 13 | 324 | 1 x 100 | 1.00 | SRR2298890 | https://sra-downloadb.be-md.ncbi.nlm.nih.gov/sos1/sra-pub-run-1/SRR2298890/SRR2298890.1 |
|  | 32℃ on day 13 | 312 | 1 x 100 | 0.96 | SRR2298891 | https://sra-downloadb.be-md.ncbi.nlm.nih.gov/sos1/sra-pub-run-1/SRR2298891/SRR2298891.1 |
|  | 27℃ on day 13 | 313 | 1 x 100 | 0.96 | SRR2298888 | https://sra-downloadb.be-md.ncbi.nlm.nih.gov/sos1/sra-pub-run-1/SRR2298888/SRR2298888.1 |
|  | 27℃ on day 13 | 306 | 1 x 100 | 0.95 | SRR2298889 | https://sra-downloadb.be-md.ncbi.nlm.nih.gov/sos1/sra-pub-run-1/SRR2298889/SRR2298889.1 |
|  | 27℃ on day 13 | 311 | 1 x 100 | 0.96 | SRR2298892 | https://sra-downloadb.be-md.ncbi.nlm.nih.gov/sos1/sra-pub-run-1/SRR2298892/SRR2298892.1 |
|  | 27℃ on day 13 | 315 | 1 x 100 | 0.97 | SRR2298893 | https://sra-downloadb.be-md.ncbi.nlm.nih.gov/sos1/sra-pub-run-1/SRR2298893/SRR2298893.1 |
| *Symbiodinium* sp. (19) | Control (0 h, 12 h/12 h day/night cycle, 25℃) | 5370 | 2 x 101 | 8.43 | DRR079242 | https://sra-downloadb.be-md.ncbi.nlm.nih.gov/sos1/sra-pub-run-2/DRR079242/DRR079242.1 |
|  | Control (48 h, 12 h/12 h day/night cycle, 25℃) | 4761 | 2 x 101 | 7.45 | DRR079243 | https://sra-downloadb.be-md.ncbi.nlm.nih.gov/sos1/sra-pub-run-2/DRR079243/DRR079243.1 |
|  | Dark condition (48 h dark period, 25℃) | 5434 | 2 x 101 | 8.53 | DRR079244 | https://sra-downloadb.be-md.ncbi.nlm.nih.gov/sos1/sra-pub-run-2/DRR079244/DRR079244.1 |
|  | Heat stress (48 h, 12 h/12 h day/night cycle, 31℃) | 5365 | 2 x 101 | 8.41 | DRR079245 | https://sra-downloadb.be-md.ncbi.nlm.nih.gov/sos1/sra-pub-run-2/DRR079245/DRR079245.1 |
|  | Heat stress in dark condition (48 h dark period, 31℃) | 4958 | 2 x 101 | 7.77 | DRR079246 | https://sra-downloadb.be-md.ncbi.nlm.nih.gov/sos1/sra-pub-run-2/DRR079246/DRR079246.1 |
| *Cladocopium* sp. (19) | Control (0 h, 12 h/12 h day/night cycle, 25℃) | 4843 | 2 x 101 | 7.67 | DRR088466 | https://sra-downloadb.be-md.ncbi.nlm.nih.gov/sos1/sra-pub-run-2/DRR088466/DRR088466.1 |
|  | Control (48 h, 12 h/12 h day/night cycle, 25℃) | 4815 | 2 x 101 | 7.63 | DRR088467 | https://sra-downloadb.be-md.ncbi.nlm.nih.gov/sos1/sra-pub-run-2/DRR088467/DRR088467.1 |
|  | Dark condition (48 h dark period, 25℃) | 5069 | 2 x 101 | 8.03 | DRR088468 | https://sra-downloadb.be-md.ncbi.nlm.nih.gov/sos1/sra-pub-run-2/DRR088468/DRR088468.1 |
|  | Heat stress (48 h, 12 h/12 h day/night cycle, 31℃) | 4256 | 2 x 101 | 6.74 | DRR088469 | https://sra-downloadb.be-md.ncbi.nlm.nih.gov/sos1/sra-pub-run-2/DRR088469/DRR088469.1 |
|  | Heat stress in dark condition (48 h dark period, 31℃) | 5543 | 2 x 101 | 8.78 | DRR088470 | https://sra-downloadb.be-md.ncbi.nlm.nih.gov/sos1/sra-pub-run-2/DRR088470/DRR088470.1 |
